# Efficacy and safety of subcutaneous mosunetuzumab plus polatuzumab vedotin in patients with relapsed/refractory large B-cell lymphoma: Japan subgroup analysis of the phase III SUNMO trial

**DOI:** 10.1007/s10147-026-03042-x

**Published:** 2026-06-15

**Authors:** Dai Maruyama, Hideki Goto, Takahiro Kumode, Keiko Aizawa, Noriko Fukuhara, Jason R. Westin, Won Seog Kim, Chiemi Mori, Tatsuki Imaizumi, Koji Kato

**Affiliations:** 1 Department of Hematology Oncology, Cancer Institute Hospital, Japanese Foundation for Cancer Research, Tokyo, Japan; 2 Division of Laboratory and Transfusion Medicine, Hokkaido University Hospital, Sapporo, Japan; 3 Department of Hematology and Rheumatology, Faculty of Medicine, Kindai University, Osaka, Japan; 4 Division of Hematology, Yamagata University Hospital, Yamagata, Japan; 5 Department of Hematology, Tohoku University, Sendai, Japan; 6 Department of Lymphoma and Myeloma, University of Texas MD Anderson Cancer Center, Houston, TX USA; 7 Department of Medicine, Division of Hematology-Oncology, Samsung Medical Center, Sungkyunkwan University School of Medicine, Seoul, Korea; 8 Chugai Pharmaceutical Co., Ltd, Tokyo, Japan; 9 Department of Hematology, Oncology, and Cardiovascular Medicine, Kyushu University Hospital, Fukuoka, Japan

**Keywords:** Aggressive non-Hodgkin lymphoma, Bispecific antibody, Japan, Large B-cell lymphoma, Mosunetuzumab, Polatuzumab vedotin

## Abstract

**Background:**

Novel, accessible treatment options, are needed for patients with relapsed/refractory (R/R) large B-cell lymphoma (LBCL). Mosunetuzumab, a T-cell–engaging bispecific antibody, plus polatuzumab vedotin, an antibody–drug conjugate, (Mosun-Pola), represents a novel fixed-duration outpatient therapy. The phase III SUNMO study (NCT05171647) demonstrated superior efficacy of Mosun-Pola versus rituximab plus gemcitabine-oxaliplatin (R-GemOx) in patients with R/R LBCL, with a manageable safety profile. We report outcomes of a Japan subgroup analysis of the SUNMO study.

**Methods:**

Patients were randomized 2:1 to receive Mosun-Pola or R-GemOx. Dual primary endpoints were centrally assessed overall response rate and progression-free survival (PFS).

**Results:**

At data cutoff (February 17, 2025), of 208 patients randomized (Mosun-Pola, *n* = 138; R-GemOx, *n* = 70), 16 were enrolled from Japan (Mosun-Pola, *n* = 9; R-GemOx, *n* = 7). All patients receiving Mosun-Pola achieved a response (100% [*n* = 9]) versus 57.1% (n = 4/7) for those receiving R-GemOx. PFS was significantly prolonged with Mosun-Pola; median PFS of 16.2 months versus 3.8 months with R-GemOx (hazard ratio: 0.42; 95% confidence interval: 0.07–2.63). In the safety-evaluable population (Mosun-Pola, *n* = 9; R-GemOx, *n* = 7), overall incidence of adverse events was similar between the Japan subgroup and the overall population across both treatment arms. Cytokine release syndrome occurred in 5 patients who received Mosun-Pola; all events were grade 1, and all resolved. No incidence of immune effector cell-associated neurotoxicity syndrome was reported.

**Conclusions:**

Mosun-Pola demonstrated promising efficacy and manageable safety in patients with R/R LBCL enrolled from Japan, which was comparable with the overall population, supporting Mosun-Pola as a potential new treatment option in this patient population.

**Clinical trial registration:**

ClinicalTrials.gov, NCT05171647.

**Supplementary Information:**

The online version contains supplementary material available at 10.1007/s10147-026-03042-x.

## Introduction

Large B-cell lymphoma (LBCL) is the most common and aggressive form of non-Hodgkin lymphoma (NHL) worldwide, and represents 45.3% of all cases of NHL in Japan [1]. Despite advances in first-line treatment, approximately 40% of patients experience refractory disease or relapse following an initial response [2, 3] and outcomes for patients for whom first-line therapy has failed remain poor [4]. Although standard of care in the second-line setting is evolving, patients who are transplant-ineligible have few treatment options, which are mainly limited to additional immunochemotherapy. Second-line treatments with a curative intent include high-dose chemotherapy with autologous stem-cell transplantation (ASCT) and chimeric antigen receptor (CAR) T-cell therapy. Both are recommended by Japanese Practical guidelines for Hematological Malignancies; high-dose chemotherapy with ASCT for medically fit patients with relapsed or refractory (R/R) LBCL who have responded to salvage therapy, or CAR T-cell therapy for patients who are refractory to first-line therapy or relapse within 1 year after achieving a complete response to first-line therapy [5, 6]. However, there are multiple challenges associated with their delivery and more than 75% of eligible patients do not receive these treatments in real-world clinical practice and outcomes for these patients are poor [7]. Barriers to the use of curative therapies may include lack of response to prior therapy, advanced age, frailty, comorbidities and/or logistical challenges. Toxicities of T-cell directed therapies, such as cytokine release syndrome (CRS), may also limit access for patients and burden healthcare systems [8]. Novel treatment options that are efficacious, have a manageable safety profile, and are accessible, are needed for patients with R/R LBCL.

Mosunetuzumab is an off-the-shelf, CD20xCD3, T-cell-engaging bispecific antibody, approved for patients with R/R follicular lymphoma after ≥ 2 prior lines of therapy [9, 10]. Polatuzumab vedotin, a CD79b targeting antibody–drug conjugate, is approved in combination with chemotherapy for patients with previously untreated or R/R diffuse LBCL (DLBCL) [11, 12]. Polatuzumab vedotin has been shown to potentiate mosunetuzumab by upregulating CD20 expression on malignant B cells and activating T-cell–dependent cytotoxicity [13], and this synergistic effect provides a potential molecular rationale for their combined use. In patients with R/R DLBCL both mosunetuzumab and polatuzumab vedotin have both individually demonstrated efficacy with tolerable safety [14, 15], and in combination have shown highly durable responses and manageable safety in a global phase II study [16, 17]. The global, randomized, phase III SUNMO trial (NCT05171647), compared mosunetuzumab in combination with polatuzumab vedotin (Mosun-Pola) versus rituximab plus gemcitabine-oxaliplatin (R-GemOx) in transplant-ineligible patients with R/R LBCL after ≥ 1 prior line of therapy [18]. At the time the SUNMO trial was designed, and until recently, R-GemOx was not approved in Japan, but globally accepted as standard of care for patients with transplant-ineligible R/R LBCL [19]. In the SUNMO trial, Mosun-Pola demonstrated clinically meaningful and statistically significant improvements in overall response rate (ORR) and progression-free survival (PFS) compared with R-GemOx, with a manageable safety profile and a low incidence of CRS events, in ASCT-ineligible patients with R/R LBCL [18].

Experience with administering bispecific antibodies (or bispecific antibodies in combination with other agents) to Japanese patients remains limited. SUNMO was the first trial in which Mosun-Pola was administered to Japanese patients; therefore to generate data in Japanese patients, we report a subgroup analysis of the SUNMO trial, to evaluate the efficacy and safety of Mosun-Pola in patients with R/R LBCL enrolled in Japan.

## Patients and methods

### Study design and patients

SUNMO was a randomized, open-label, multicenter phase III study evaluating efficacy and safety of Mosun-Pola compared with R-GemOx in patients with R/R LBCL. Here, we focus on the efficacy and safety of Mosun-Pola in a Japan subgroup analysis of patients with R/R LBCL included in the study (subgroup was pre-defined). The protocol was approved by institutional review boards or ethics committees; the study was conducted in accordance with the Declaration of Helsinki, International Conference on Harmonization Guidelines for Good Clinical Practice, and applicable laws and regulations. All patients provided written informed consent.

Full study details have been published previously [18]. Briefly, eligible patients were ≥ 18 years with histologically confirmed LBCL, including DLBCL not otherwise specified (NOS), high-grade B-cell lymphoma (HGBCL; NOS or double/triple hit), follicular lymphoma (FL) grade 3b, or transformed FL and an Eastern Cooperative Oncology Group performance status of 0–2. Patients had received ≥ 1 prior systemic anti-lymphoma therapy and must have been ineligible for ASCT.

Patients were randomized 2:1 to Mosun-Pola or R-GemOx and stratified by number of prior lines of systemic therapy (1 vs. ≥ 2) and outcome after last systemic therapy (relapsed vs. refractory). Patients in the Mosun-Pola group received 8 cycles (21-day cycles) of subcutaneous (SC) mosunetuzumab without the need for hospitalization: 5 mg on Day 1, 45 mg on Day 8 and Day 15 of Cycle 1, and 45 mg on Day 1 of Cycles 2–8. Corticosteroid premedication was required only during Cycle 1 prior to each mosunetuzumab dose. Polatuzumab vedotin was administered intravenously for 6 cycles (21-day cycles) at 1.8 mg/kg on Day 1 of Cycles 1–6. Patients in the R-GemOx group received 8 cycles of standard rituximab (375 mg/m^2^), gemcitabine (1000 mg/m^2^), and oxaliplatin (100 mg/m^2^) intravenously on Day 1 of Cycles 1–8 (14-day cycles, which could be extended to 21 days if needed due to hematologic toxicity).

### Endpoints and outcome assessments

Dual primary endpoints were ORR and PFS, both centrally assessed per Lugano criteria [20], and a key secondary endpoint was overall survival (OS). Adverse events (AEs) were assessed using the National Cancer Institute Common Toxicity Criteria for Adverse Events (Version 5.0) and CRS was assessed using the American Society for Transplantation and Cellular Therapy grading criteria [21].

### Statistical analysis

Overall type I error rate was controlled at 5% (two-sided) using hierarchical testing including possible recycling to adjust for multiple statistical testing. ORR was compared between treatment arms in the interim analysis population, which was defined as the first 178 patients randomized (data cutoff: April 19, 2024), using stratified Cochran–Mantel–Haenszel testing (2.5% two-sided α-level); 95% confidence intervals (CIs) for difference in ORR were constructed using the Hauck Andersen method, and 95% CIs for ORR were calculated using the Clopper-Pearson method. Stratification factors used in the efficacy analyses included the number of previous lines of systemic therapy for aggressive lymphoma (1 vs. ≥ 2) and outcome after the last systemic therapy (relapsed vs. refractory). PFS was compared in the intention-to-treat population, defined as all patients randomized (data cutoff: February 17, 2025) using a stratified log-rank test with a two-sided 0.05 level if the ORR primary analysis was positive or 0.025 level if the ORR primary analysis was negative. The Kaplan–Meier method was used to estimate median PFS, if reached, and PFS distribution for each treatment arm, and Brookmeyer-Crowley methodology to construct the 95% CI for median PFS for each treatment arm. For PFS and OS, a stratified Cox proportional-hazards model was used to estimate the hazard ratio (HR) and corresponding 95% CIs. The key secondary endpoint of OS was evaluated as an interim analysis using the Lan-DeMets α-spending function with an O’Brien-Fleming boundary to control for type I error. The safety-evaluable population included patients who received at least 1 dose of treatment.

## Results

### Patients

At data cutoff (February 17, 2025), 208 patients were randomized to Mosun-Pola (*n* = 138) and R-GemOx (*n* = 70); of these, 16 were enrolled from Japan (Mosun-Pola, *n* = 9; R-GemOx, *n* = 7). Baseline demographic and clinical characteristics were comparable between the Japan subgroup and the overall population except for the incidence of HGBCL, which was higher in the Mosun-Pola arm of the Japan subgroup (Table 1). In the Japan subgroup, median age was 73 years (range, 57–79) in the Mosun-Pola group and 71 years (range, 65–78) in the R-GemOx group, and 55.6% (*n* = 5) and 100% (*n* = 7) of patients were aged ≥ 65 years, respectively.

**Table 1:** Baseline demographic and clinical characteristics

*n* (%) unless stated	Japan subgroup		Overall population	
	Mosun-Pola *n* = 9	R-GemOx *n* = 7	Mosun-Pola *n* = 138	R-GemOx *n* = 70
Median age, years (range)	73 (57–79)	71 (65–78)	62 (23‒87)	63 (29‒85)
≥ 65 years	5 (55.6)	7 (100)	54 (39.1)	32 (45.7)
Male	6 (66.7)	3 (42.9)	76 (55.1)	45 (64.3)
ECOG performance status				
0	7 (77.8)	5 (71.4)	69 (50.0)	40 (57.1)
1	1 (11.1)	2 (28.6)	51 (37.0)	29 (41.4)
2	1 (11.1)	0	18 (13.0)	1 (1.4)
Ann Arbor stage at study entry				
I/II	2 (22.2)	1 (14.3)	34 (24.6)	14 (20.0)
III/IV	7 (77.8)	6 (85.7)	104 (75.4)	56 (80.0)
IPI score at study entry				
0‒2	5 (55.6)	2 (28.6)	67 (48.6)	36 (51.4)
3‒5	4 (44.4)	5 (71.4)	71 (51.4)	34 (48.6)
Bulky disease				
> 7.5 cm	2 (22.2)	1 (14.3)	52 (37.7)	19 (27.1)
> 10 cm	1 (11.1)	0	28 (20.3)	5 (7.1)
Non-Hodgkin lymphoma subtype				
DLBCL	4 (44.4)	6 (85.7)	109 (79.0)	54 (77.1)
HGBCL	5 (55.6)	1 (14.3)	26 (18.8)	14 (20.0)
FL grade 3b	0	0	3 (2.2)	2 (2.9)
Transformed FL (as part of DLBCL and HGBCL)	3/9 (33.3)	1/7 (14.3)	17/135 (12.6)	6/68 (8.8)
Cell-of-origin^a^	(*n* = 9)	(*n* = 7)	(*n* = 135)	(*n* = 68)
GCB	6 (66.7)	4 (57.1)	56 (41.5)	20 (29.4)
ABC	2 (22.2)	3 (42.9)	42 (31.1)	27 (39.7)
Unclassified	0	0	15 (11.1)	5 (7.4)
Missing	1 (11.1)	0	22 (16.3)	16 (23.5)
Number of prior lines of therapy				
Median (range)	1 (1–4)	1 (1–4)	2 (1–9)	2 (1–5)
1	5 (55.6)	4 (57.1)	61 (44.2)	30 (42.9)
≥ 2	4 (44.4)	3 (42.9)	77 (44.2)	40 (57.1)
Previous CAR T-cell therapy	0	0	3 (2.2)	5 (7.1)
R/R status				
Primary R/R within 12 months of first prior therapy	6 (66.7)	3 (42.9)	100 (72.5)	53 (75.7)
Refractory to last prior therapy	5 (55.6)	3 (42.9)	97 (70.3)	48 (68.6)

Clinical cutoff date: February 17, 2025

*ABC* activated B-cell-like, *CAR* chimeric antigen receptor, *DLBCL* diffuse large B-cell lymphoma, *ECOG* Eastern Cooperative Oncology Group, *FL* follicular lymphoma, *GCB* germinal center B-cell-like, *HGBCL* high-grade B-cell lymphoma, *IPI* International Prognostic Index, *Mosun-Pola* mosunetuzumab in combination with polatuzumab vedotin, *R-GemOx* rituximab plus gemcitabine-oxaliplatin, *R/R* relapsed/refractory

^a^Centrally assessed

In the Mosun-Pola and R-GemOx groups, 55.6% (*n* = 5) and 42.9% (*n* = 3) had primary refractory disease, and 44.4% (*n* = 4) and 71.4% (*n* = 5) had an International Prognostic Index score of 3–5, respectively. The median number of prior lines of therapy received was 1 (range, 1–4) in both treatment arms; no patients had received prior CAR T-cell therapy. Similar to the Mosun-Pola group in the overall population, the median number of treatment cycles received in the Japan subgroup was 8 (range, 3–8) for mosunetuzumab and 6 (range, 3–6) for polatuzumab vedotin (Supplementary Table 1). In the R-GemOx group, the median number of treatment cycles received in the Japan subgroup was 5 (1–8) for rituximab, gemcitabine and oxaliplatin.

### Efficacy

**Fig. 1:**
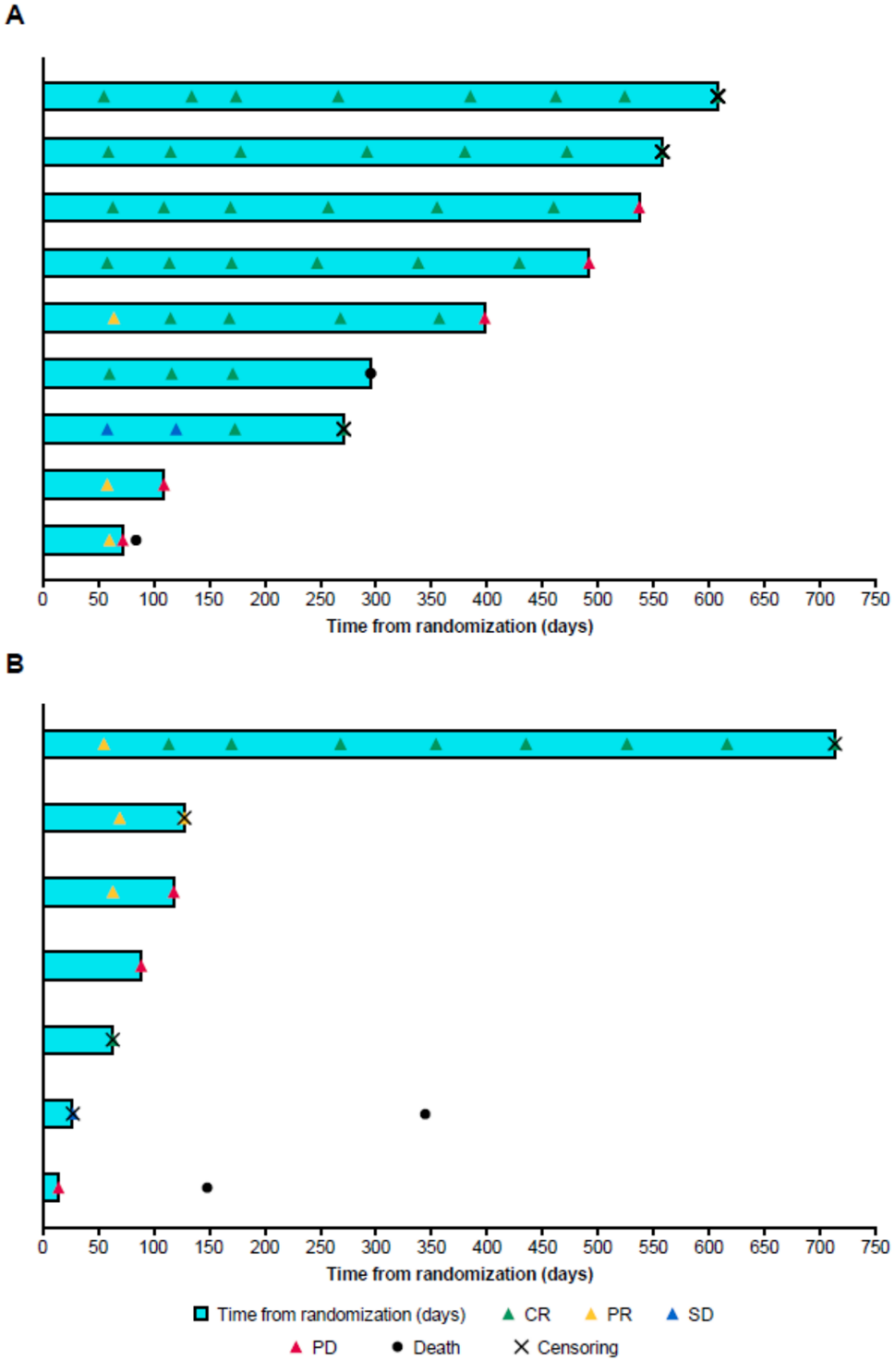
Duration of response **A** Mosun-Pola, **B** R-GemOx. *CR* complete response, *PD* disease progression, *PR* partial response, *SD* stable disease

Consistent with the overall population, the Japan subgroup showed an improvement with Mosun-Pola over R-GemOx in the dual primary endpoints of ORR and PFS at each primary analysis. Of note, in the overall population, the primary endpoint of ORR was met at the time of the interim analysis and the primary endpoint of PFS was met at the primary analysis. The independent data monitoring committee subsequently recommended early closure of enrollment at 208 patients of the 222 originally planned. Among patients enrolled in Japan, all those in the Mosun-Pola group (100%, *n* = 9) achieved a response, compared with 57.1% (*n* = 4/7) of patients in the R-GemOx group (Table 2). The complete response rate in the Mosun-Pola group was 77.8% (*n* = 7/9) compared with 28.6% (*n* = 2/7) in the R-GemOx group (Table 2). Most patients achieved an initial response within 3–4 months (Fig. 1A, B) [18].

**Table 2:** Response rates

*n* (%), [95% CI]	Japan subgroup		Overall population	
	Mosun-Pola *n* = 9	R-GemOx *n* = 7	Mosun-Pola *n* = 138	R-GemOx *n* = 70
Overall response	9 (100) [66.4–100.0]	4 (57.1) [18.4–90.1]	97 (70.3) [61.9–77.8]	28 (40.0) [28.5–52.4]
Complete response	7 (77.8) [40.0–97.2]	2 (28.6) [3.7–71.0]	71 (51.4) [42.8–60.0]	17 (24.3) [14.8–36.0]
Partial response	2 (22.2) [2.8–60.0]	2 (28.6) [3.7–71.0]	26 (18.8) [12.7–26.4]	11 (15.7) [8.1–26.4]
Stable disease	0 [0.0–33.6]	1 (14.3) [0.4–57.9]	15 (10.9) [6.2–17.3]	15 (21.4) [12.5–32.9]
Disease progression	0 [0.0–33.6]	2 (28.6) [3.7–71.0]	19 (13.8) [8.5–20.7]	19 (27.1) [17.2–39.1]

*CI* confidence interval, *Mosun-Pola* mosunetuzumab in combination with polatuzumab vedotin, *R-GemOx* rituximab plus gemcitabine-oxaliplatin

**Fig. 2:**
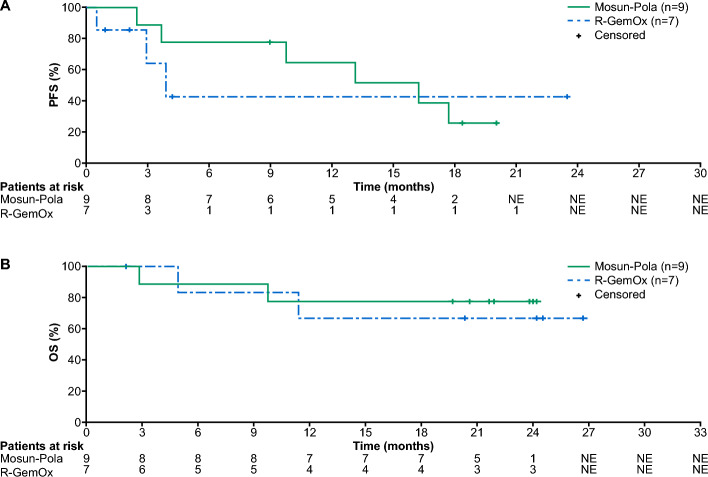
Kaplan–Meier estimates (ITT population) of **A** centrally-assessed PFS in the primary analysis and **B** interim analysis of OS. *ITT* intention-to-treat, *Mosun-Pola* mosunetuzumab in combination with polatuzumab vedotin, *NE* not estimable, *OS* overall survival, *PFS* progression-free survival, *R-GemOx* rituximab plus gemcitabine-oxaliplatin

Mosun-Pola reduced the risk of death or progression by 58% versus R-GemOx, with a median PFS of 16.2 months (95% CI 9.7–not estimable [NE]) versus 3.8 months (95% CI 2.9–NE), respectively (HR: 0.42; 95% CI 0.07–2.63; Fig. 2A). In the overall population, median PFS was significantly greater for Mosun-Pola compared with R-GemOx (11.5 months vs. 3.8 months; HR: 0.41 (95% CI 0.3–0.6; *p* < 0.0001) [18].

At the interim analysis of OS, median OS was not reached for both treatment arms (Fig. 2B). Although patient numbers in the Japan subgroup were small, OS numerically favored Mosun-Pola versus R-GemOx (HR: 0.76; 95% CI 0.10–5.51). At the interim analysis of the overall population, median OS was 18.7 months for Mosun-Pola and 13.6 months for R-GemOx (HR: 0.80; 95% CI 0.5–1.2; *p* = 0.2835).

### Safety

In the safety-evaluable population (Mosun-Pola, *n* = 9; R-GemOx, *n* = 7), the overall incidence of AEs was similar between the Japan subgroup and the overall population across both treatment arms (Table 3). In the Japan subgroup, treatment-related serious AEs occurred in 2 patients (1 in each group) and no patients discontinued Mosun-Pola due to an AE.

**Table 3:** Safety summary

*n* (%), unless stated	Japan subgroup		Overall population	
	Mosun-Pola *n* = 9	R-GemOx *n* = 7	Mosun-Pola *n* = 135	R-GemOx *n* = 64
Any AEs	9 (100)	7 (100)	131 (97.0)	61 (95.3)
Any treatment-related AEs	9 (100)	7 (100)	126 (93.3)	57 (89.1)
Serious AEs	2 (22.2)	1 (14.3)	45 (33.3)	16 (25.0)
Any treatment-related serious AEs	1 (11.1)	1 (14.3)	33 (24.4)	13 (20.3)
Grade 3–5 AEs	7 (77.8)	6 (85.7)	86 (63.7)	41 (64.1)
Treatment-related grade 3–5 AEs	7 (77.8)	5 (71.4)	73 (54.1)	35 (54.7)
Grade 5 AEs	1 (11.1)^a^	0	7 (5.2)^b^	4 (6.3)^b^
Mosun-related grade 5 AEs	1 (11.1)	–	2 (1.5)	–
Pola-related grade 5 AEs	1 (11.1)	–	2 (1.5)	–
R-GemOx-related grade 5 AEs	–	0	–	2 (3.1)
AE leading to any study drug discontinuation	0	1 (14.3)^c^	3 (2.2)^d^	3 (4.7)^d^
Mosun discontinuation	0	–	2 (1.5)	–
Pola discontinuation	0	–	3 (2.2)	–
R-GemOx discontinuation	–	1 (14.3)	–	3 (4.7)
AE leading to any study drug dose modification	0	2 (28.6)	5 (3.7)	5 (7.8)
Mosun modification	0	–	1 (0.7)	–
Pola modification	0	–	4 (3.0)	–
R-GemOx modification	–	2 (28.6)	–	5 (7.8)
AE leading to any study drug dose interruption	6 (66.7)	5 (71.4)	61 (45.2)	32 (50.0)
Mosun interruption	6 (66.7)	–	60 (44.4)	–
Pola interruption	4 (44.4)	–	54 (40.0)	–
R-GemOx interruption	–	5 (71.4)	–	32 (50.0)

Clinical cutoff date: February 17, 2025

*AE* adverse event, *COVID-19* coronavirus disease-19, *Mosun* mosunetuzumab, *Mosun-Pola* mosunetuzumab in combination with polatuzumab vedotin, *Pola* polatuzumab vedotin, *R-GemOx* rituximab plus gemcitabine-oxaliplatin

^a^Mosun-Pola: COVID-19 pneumonia (*n* = 1)

^b^Mosun-Pola: pulmonary embolism (*n* = 1), septic shock (*n* = 1), cardiac arrest (*n* = 1), cytomegalovirus infection reactivation (*n* = 1), COVID-19 (*n* = 1), and COVID-19 pneumonia (*n* = 2); R-GemOx: pneumonia (*n* = 1), septic shock (*n* = 1), sepsis (*n* = 1), and COVID-19 pneumonia (*n* = 1)

^c^R-GemOx: delirium (*n* = 1)

^d^Mosun-Pola withdrawn due to pneumonitis (*n* = 1) and cytomegalovirus infection reactivation (*n* = 1); only Pola withdrawn due to infusion-related reaction (*n* = 1); R-GemOx withdrawn due to delirium (*n* = 1), embolism (*n* = 1) and respiratory syncytial virus infection (*n* = 1)

CRS occurred in 5 patients who received Mosun-Pola (Supplementary Table 2); all events were grade 1 and all resolved. Median time to first onset of CRS was 3 days (range, 2–5) from the most recent mosunetuzumab dose and median duration of CRS was 3 days (range, 1–4). One patient received corticosteroids for CRS management; no patients received tocilizumab. No incidence of immune effector cell-associated neurotoxicity syndrome (ICANS) was reported.

Any grade infections occurred in 55.6% (*n* = 5) and 28.6% (*n* = 2) of patients in the Mosun-Pola and R-GemOx groups, respectively (Supplementary Table 3). One grade 5 event (COVID-19 pneumonia) occurred in a patient following completion of study treatment with Mosun-Pola. Rates of serious infections were low (1 patient in each treatment arm). AEs of special interest are summarized in Table 4; grade ≥ 3 neutropenia occurred in 55.6% (*n* = 5) of patients in the Mosun-Pola group and 57.1% (*n* = 4) in the R-GemOx group and 1 patient in the R-GemOx group experienced febrile neutropenia. Peripheral neuropathy occurred in 11.1% (*n* = 1) and 42.9% (*n* = 3) of patients in the Mosun-Pola and R-GemOx groups, respectively.

**Table 4:** AEs of special interest and other selected AEs

*n* (%), unless stated	Japan subgroup		Overall population	
	Mosun-Pola *n* = 9	R-GemOx *n* = 7	Mosun-Pola *n* = 135	R-GemOx *n* = 64
Thrombocytopenia, any grade	0	5 (71.4)	12 (8.9)	42 (65.6)
Grade ≥ 3	0	3 (42.9)	3 (2.2)	23 (35.9)
Neutropenia^a^, any grade	5 (55.6)	4 (57.1)	62 (45.9)	35 (54.7)
Grade ≥ 3	5 (55.6)	4 (57.1)	45 (33.3)	20 (31.3)
Febrile neutropenia	0	1 (14.3)	3 (2.2)	2 (3.1)
Neurologic AE^b^, any grade	5 (55.6)	4 (57.1)	61 (45.2)	34 (53.1)
Grade ≥ 3	0	0	2 (1.4)	2 (3.1)
Anemia, any grade	3 (33.3)	4 (57.1)	41 (30.4)	27 (42.2)
Grade ≥ 3	1 (11.1)	3 (42.9)	8 (5.9)	12 (18.8)
Peripheral neuropathy (SMQ), any grade	1 (11.1)	3 (42.9)	33 (24.4)	27 (42.2)
Grade ≥ 3	0	0	0	0
Pneumonitis/interstitial lung disease, any grade	0	0	7 (5.2)	0
Grade ≥ 3	0	0	3 (2.2)	0
Rash, any grade	3 (33.3)	0	23 (17.0)	4 (6.3)
Grade ≥ 3	1 (11.1)	0	1 (0.7)	0
Tumor flare, any grade	0	0	9 (6.7)	0
Grade ≥ 3	0	0	2 (1.5)	0
Tumor lysis syndrome, any grade	0	0	1 (0.7)	0
Grade ≥ 3	0	0	1 (0.7)	0
Infusion-related reaction, any grade	1 (11.1)	2 (28.6)	10 (7.4)	13 (20.3)
Grade ≥ 3	0	0	2 (1.5)	1 (1.6)
Secondary malignancies, any grade	0	0	2 (1.5)	1 (1.6)
Grade ≥ 3	0	0	1 (0.7)	0

*AE* adverse event, *AEGT* adverse event group terms, *Mosun-Pola* mosunetuzumab in combination with polatuzumab vedotin, *PT* preferred term, *R-GemOx* rituximab plus gemcitabine-oxaliplatin, *SOC* System organ class, *SMQ* Standardised MedDRA Query

^a^Includes neutrophil count decreased and neutropenia

^b^Neurologic AEs include AEs reported as primary or secondary PTs in either the SOC “Nervous system disorders” or SOC “Psychiatric disorders” as defined by the AEGT “CD20CD3 Neurotoxicity-Original 2018”

## Discussion

Patients with R/R LBCL who do not receive standard second-line treatments with a curative intent have poor outcomes. In this subgroup analysis of the phase III SUNMO trial, Mosun-Pola demonstrated promising efficacy and manageable safety in patients with R/R LBCL enrolled in Japan, which was comparable with the overall population [18]. Mosun-Pola provided a 58% reduction in the risk of death or progression compared with R-GemOx, and all patients receiving Mosun-Pola achieved a response, with 77.8% achieving a complete response. The safety profile was manageable and in line with the known profiles of the individual agents. The overall incidence of AEs across both treatment arms was similar between the Japan subgroup and the overall population. CRS events occurred in 56% of patients receiving Mosun-Pola and all events were grade 1 and manageable.

High durable response rates have been shown in patients with LBCL treated with other T-cell–engaging therapies, including the bispecific antibody epcoritamab (third-line or later) and CAR T-cell therapies axicabtagene ciloleucel (second-line or later), tisagenlecleucel (third-line or later), and lisocabtagene maraleucel (second-line or later). In the subgroup analyses of Japanese patients with R/R LBCL from these studies, overall response rates of 70–87% with CAR T-cell therapies and 56% with epcoritamab, and complete response rates of 27–56% and 47%, respectively, were observed [22–25]. These are comparable with the response rates observed in the overall populations of patients with R/R LBCL, with ORR of 52–83% with CAR T-cell therapies and 63% with epcoritamab, and corresponding complete response rates of 40–65% and 40%, respectively [26–29]. Furthermore, in the subgroup analyses of Japanese patients, any grade and grade ≥ 3 CRS rates of 50–81% and 0–22% were reported for CAR T-cell therapies, and 83% and 8% for epcoritamab, respectively [22–25], which tended to be slightly higher than the rates observed with Mosun-Pola combination therapy in this analysis (56% and 0%, respectively). However, it should be noted that cross-trial comparisons are challenging due to differences in patient characteristics and duration of follow-up, and in all subgroup studies cited, patient numbers were low; therefore, any conclusions should be viewed with caution.

CAR T-cell therapy is an effective treatment option for many patients with R/R LBCL; however, several barriers preclude eligible patients from receiving treatment, including logistical challenges, manufacturing limitations, and toxicity concerns [8]. Mosun-Pola is the first bispecific antibody and antibody–drug conjugate combination to demonstrate significantly improved efficacy and favorable safety with lower rates of peripheral neuropathy and other chemotherapy-related toxicities [18]. Therefore, Mosun-Pola offers a practical alternative as a fixed duration, off-the-shelf regimen, with a low incidence of CRS and no incidence of ICANS events that can be delivered in an outpatient setting without the use of conventional chemotherapy.

A strength of the study includes the enrollment of patients with high-risk disease characteristics; over 60% of patients were primary refractory or had relapsed within 12 months of their first prior therapy and 55.6% had HGBCL. A limitation of the study includes the small sample size. In addition, longer follow up is needed to confirm the results.

## Conclusion

In this subgroup analysis of the SUNMO trial, Mosun-Pola demonstrated promising efficacy and manageable safety in patients with R/R LBCL enrolled from Japan, which was comparable with the overall population, and supports Mosun-Pola as a potential new treatment option in this patient population.

## Supplementary Information

Below is the link to the electronic supplementary material.

Supplementary file1 (DOCX 39 KB)

## Data Availability

Qualified researchers may request access to individual patient-level data through the clinical study data request platform (www.clinicalstudydatarequest.com). For further details on Chugai’s Data Sharing Policy and how to request access to related clinical study documents, see www.chugai-pharm.co.jp/english/profile/rd/ctds_request.html.
