# Supplementary material for: Efficacy and safety of subcutaneous mosunetuzumab plus polatuzumab vedotin in patients with relapsed/refractory large B-cell lymphoma: Japan subgroup analysis of the phase III SUNMO trial
Source: Int J Clin Oncol. 2026 Jun 15;31(8):1547–56. doi: 10.1007/s10147-026-03042-x (PMC13401542; doi:10.1007/s10147-026-03042-x)
Supplement: Supplementary file 1 — Supplementary file1 (DOCX 39 KB) [file 10147_2026_3042_MOESM1_ESM.docx]

**Supplementary Appendix**

**Supplementary Table 1** Study drug exposure

|  | Japan subgroup | Overall population |
| --- | --- | --- |
|  | *n*=9 | *n*=135 |
| **Mosunetuzumab** | | |
| Median treatment duration, days (range) | 159 (43–197) | 148 (1–221) |
| Median number of cycles (range) | 8 (3–8) | 8 (1–8) |
| Dose intensity, % |  |  |
| Median (range) | 93.1 (76.0–100.0) | 98.7 (20.0–104.8) |
| ≥90%, *n* (%) | 6 (66.7) | 106 (78.5) |
| **Polatuzumab vedotin** | | |
| Median treatment duration, days (range) | 106 (43–155) | 106 (1–155) |
| Median number of cycles (range) | 6 (3–6) | 6 (1–6) |
| Dose intensity, % |  |  |
| Median (range) | 99.5 (68.4–101.9) | 97.9 (53.7–111.0) |
| ≥90%, *n* (%) | 6 (66.7) | 105 (77.8) |
|  | *n*=7 | *n*=64 |
| **Rituximab** | | |
| Median treatment duration, days (range) | 59.0 (1–106) | 75 (1–155) |
| Median number of cycles (range) | 5 (1–8) | 5 (1–8) |
| Dose intensity, % |  |  |
| Median (range) | 93.0 (54.6–109.8) | 90.4 (49–109.8) |
| ≥90%, *n* (%) | 4 (57.1) | 33 (51.6) |
| **Gemcitabine** | | |
| Median treatment duration, days (range) | 58.0 (1–106) | 74.5 (1–155) |
| Median number of cycles (range) | 5 (1–8) | 5 (1–8) |
| Dose intensity, % |  |  |
| Median (range) | 98.3 (54.9–100.5) | 94.7 (51.7–130.5) |
| ≥90%, *n* (%) | 5 (71.4) | 36 (56.3) |
| **Oxaliplatin** | | |
| Median treatment duration, days (range) | 58.0 (1–106) | 74.5 (1–155) |
| Median number of cycles (range) | 5 (1–8) | 5 (1–8) |
| Dose intensity |  |  |
| Median (range) | 93.0 (52.1–100.2) | 91.7 (50.1–130.5) |
| ≥90%, *n* (%) | 4 (57.1) | 33 (51.6) |

**Supplementary Table 2** Summary of CRS events and management in the Mosun-Pola arm

| Patients with ≥1 CRS AE, n (%)  unless stated | Japan subgroup  *n*=9 | Overall population  *n*=135 |
| --- | --- | --- |
| **Any grade** | 5 (55.6) | 35 (25.9) |
| Grade 1 | 5 (55.6) | 29 (21.5) |
| Grade 2 | 0 | 5 (3.7) |
| Grade 3 | 0 | 1 (0.7) |
| **Any serious event of CRS^a^** | 0 | 7 (5.2) |
| **Median onset to first CRS event, days (range)** | 3 (2–5) | 3 (1–6) |
| **Median duration, days (range)** | 3 (1–4) | 3 (1–11) |
| **CRS management** |  |  |
| Tocilizumab | 0 | 6 (4.4) |
| Corticosteroids | 1 (11.1) | 5 (3.7) |

^a^Events that required new or prolonged hospitalization

AE, adverse event; CRS, cytokine release syndrome; Mosun-Pola, mosunetuzumab in combination with polatuzumab vedotin

**Supplementary Table 3** Incidence of infections and infestations

| *n* (%), unless stated | Japan subgroup | | Overall population | |
| --- | --- | --- | --- | --- |
|  | Mosun-Pola  *n*=9 | R-GemOx  *n*=7 | Mosun-Pola  *n*=135 | R-GemOx  *n*=64 |
| **Any grade AE** | 5 (55.6) | 2 (28.6) | 69 (51.1) | 20 (31.3) |
| Grade 1 | 1 (11.1) | 0 | 9 (6.7) | 4 (6.3) |
| Grade 2 | 3 (33.3) | 1 (14.3) | 39 (28.9) | 7 (10.9) |
| Grade 3 | 0 | 1 (14.3) | 15 (11.1) | 5 (7.8) |
| Grade 4 | 0 | 0 | 1 (0.7) | 0 |
| Grade 5^a^ | 1 (11.1) | 0 | 5 (3.7) | 4 (6.3) |
| Serious AE | 1 (11.1) | 1 (14.3) | 22 (16.3) | 9 (14.1) |
| **Mosun withdrawn due to AE** | 0 | 0 | 1 (0.7) | 0 |
| **Resolved non-fatal AEs** | 4/4 (100) | 2/2 (100) | 58/67 (86.6) | 16/19 (84.2) |
| **Median time to onset from Cycle 1 Day 1, days (range)** | 64 (37–229) | 6.5 (6–7) | 51 (1–229) | 47 (4–206) |
| **Median duration, days (range)** | 15 (8–65) | 22 (15–211) | 12 (2–407) | 8 (2–211) |

^a^Grade 5 AEs in Mosun-Pola arm: septic shock (*n*=1), COVID-19 pneumonia (*n*=2, including 1 Japanese patient), COVID-19 (*n*=1), cytomegalovirus infection reactivation (*n*=1); in R-GemOx arm: pneumonia (*n*=1), septic shock (*n*=1), sepsis (*n*=1), COVID-19 pneumonia (*n*=1)

AE, adverse event; Mosun-Pola, mosunetuzumab in combination with polatuzumab vedotin; R-GemOx, rituximab plus gemcitabine-oxaliplatin
